# Employment of Female Assistant Physicians in Female Wards of Insane Asylums

**Published:** 1879-05

**Authors:** 


### Editorial.

### Employment of Female Assistant Physicians, in the Female Wards of Insane Asylums.

A bill has been introduced in the Assembly of the State of New York, requiring the trustees of the four State asylums for the insane "to employ one or more competent and well educated female physicians to have charge of the female patients of said asylum, under the direction of the medical superintendents of the several asylums, as in the case of the other or male assistant physicians, and to take the place of such male assistant physician or physicians in the wards of female patients."

The exception taken to this bill by physicians in Buffalo is embraced in the following resolutions passed by the Buffalo Medical Association, with the reasons therefor:

*Resolved*, That this Association emphatically protest against the adoption of any such enactment for the reasons:

First. That it has not been shown that male assistant physicians are in any way incompetent to deal with the various maladies incident to insanity in women.

Second. That female physicians by virtue of their sex and the peculiar conditions incident thereto, would at times be incompetent to assume the duties of the position.

Third. That a mixed Medical Staff would be wholly subversive of good discipline and the proper and scientific management of institutions for the Insane where both sexes are treated, and that the Superintendents and Managers of the State Insane Asylums are, in our opinion, fully competent to suggest and select those who are to act as Assistants

Fouth. The impropriety, unfairness and danger of class legislation in professional, scientific or religious associations or interests.

*Resolved*—That this Association earnestly requests the members of the Legislature from the City of Buffalo and the County of Erie to do all in their power to defeat any such unwise and revolutionary measure.

Dr. H. B. Wilbur, in a letter to the Editor of the Syracuse *Courier*, answering objections to this bill, says of these resolutions:

"Inasmuch as one of the active members of the Association is a co-trustee with Dr. Gray, in the new Insane Asylum at Buffalo, and two of the others bear the same name, as one of his Medical Assistants, perhaps personal influences were unconsciously operative in the passage of the resolutions. At all events, the somewhat presumptuous and bigoted opinions they utter may be fairly offset by the abundant approval of the measure at the hands of medical men of equal standing in the profession, but with more liberal views."

And he also uses the following infamously scandalous argument in favor of the bill:

"In some of the lunatic wards of our County Poor-houses male attendants have had the charge and care of the female patients; have had the keys that gave access to their rooms; insane women have given birth to children, begotten in the house. So it is said, though no mention is made of it in the reports of the Commissioner of Lunacy.

"If this were true, would there be any impropriety in passing a law to require the managers of such institutions to employ female attendants, in the care of female patients?"

Does Dr. Wilbur soberly propose to intimate that the bastard children of our insane pauper women can in any way be traced to the male attendants in Asylums? Does he not well know that these children come by mail (male)—grow spontaneously—spring up from behind fences or other ambuscade, and are in no way to be expected from common causes? For a physician to intimate any knowledge of *where* or *how* these pauper children are begotten is so absurd as to require no reply.

In his letter he seems to trace our Association resolutions to Utica as the chief source of our inspiration, and we have no objection to the alleged source, but, for the benefit of our old friend Dr. Wilbur, we assure him the inspiration of the fourth reason was direct from God himself, through the editor of this journal; and though he *has had* a nephew in the Utica Asylum, neither the young Doctor or Utica Asylum ever urged this objection to legislative action in this connection.

Women as Doctors, possibly as Assembly(wo)men and Senatoresses (?) may yet be found to be important as a general matter of economy and reform. We personally favor the women, and wonder how—when the choice was left wholly to Superintendents of Asylums—they should have so strangely neglected their privileges. Of course female physicians are a necessity in female wards, and quite likely would be found even better as assistants, and *possibly as Superintendents* in *all* public institutions.

The provision for and care of the insane in the State of New York is a subject open for consideration, and we expect to see the present system undergo important changes.

